# Health Service Use and Treatment Choices for Pediatric Eosinophilic Esophagitis: Findings From a Cross-Sectional Survey of Australian Carers

**DOI:** 10.3389/fped.2020.00147

**Published:** 2020-04-17

**Authors:** Nicole Hannan, Amie Steel, Sara S. McMillan, Evelin Tiralongo

**Affiliations:** ^1^School of Pharmacy and Pharmacology, Griffith University, Southport, QLD, Australia; ^2^Faculty of Health, Australian Research Centre in Complementary and Integrative Medicine, University of Technology Sydney, Ultimo, NSW, Australia

**Keywords:** complementary medicine, therapy, allergy, child, EoE

## Abstract

**Objectives:** The incidence and the prevalence of eosinophilic esophagitis (EoE) are increasing, and healthcare utilization among children with EoE is high. This study provides novel insights into the health services and the treatments, including complementary medicines (CMs), used by carers to manage their children's EoE as well as the carers' beliefs and attitudes toward these treatments.

**Methods:** A national cross-sectional online survey was conducted in Australia between September 2018 and February 2019. The survey included questions about health service and treatment utilization, health insurance and government support, health-related quality of life of children with EoE and their carers, views and attitudes toward CM use, and perceived efficacy of treatment.

**Results:** The survey was completed by 181 carers (96.6% of whom were mothers) of EoE children. Most children (91.2%, *n* = 165) had seen a medical doctor for their EoE, and almost half had consulted with a CM practitioner (40.3%, *n* = 73). Pharmaceuticals (*n* = 156, 86.2%) were the most commonly used treatment option, followed by dietary changes (*n* = 142, 78.5%), CM products (*n* = 109, 60.2%), and CM therapies (*n* = 42, 23.2%). Most children received care from numerous practitioners on multiple occasions, indicating a substantial financial and treatment-related burden.

**Conclusions:** A variety of practitioners are involved in the care of children with EoE, and a high rate of CM use warrants further attention to ensure that appropriate treatment is provided. Carer involvement and guidance, combined with individual practitioner expertise, referrals, and collaboration between providers, is essential to successfully navigate this complex disease and provide adequate care for these patients.

## Introduction

Eosinophilic esophagitis (EoE) is a rare antigen-driven inflammatory gastrointestinal disorder characterized by elevated levels of eosinophils in the esophagus, esophageal dysfunction, and gastrointestinal symptoms (1). EoE incidence is increasing globally, with an estimated prevalence of one in every 2,000 people (2–4). International clinical guidelines list EoE first-line treatment options as proton-pump inhibitors, swallowed topical steroids, elimination diets, and elemental formula (5–8). While there are no national guidelines in Australia for the management of EoE, international guidelines are usually applied (9).

Given the high frequency of healthcare utilization among children with EoE (10), it is important to better understand the patterns of use in order to improve the support for these children and their carers as well as facilitate more coordinated and collaborative care between healthcare providers. Research suggests that complementary medicine (CM), a diverse range of medical and healthcare practices and products not currently regarded as part of conventional medicine (11), may be included in the range of healthcare accessed by carers of children diagnosed with gastroenterological conditions in Australia (12) and abroad (13). In order to address EoE symptoms, carers may choose CMs for their children, under the assumption that CMs are safe (14, 15); however, to our knowledge, no research has examined all healthcare accessed for pediatric EoE, including CM. In response, this study is the first to describe the health services and the treatments, including CMs, used by carers to manage their children's EoE as well as the carers' beliefs and attitudes toward these treatments.

## Methods

### Definitions

CM involves two broad classifications, defined in this study as either CM products (i.e., probiotics) or CM therapies (i.e., massage) (11).

### Study Design and Setting

A national cross-sectional online survey was conducted between September 2018 and February 2019. Ethics approval was obtained from the Griffith University Human Research Ethics Committee (#2018/120). The survey included the following domains: demographics; health service and treatment utilization; health insurance, government support, and rebates; health-related quality of life (HRQoL) of children with EoE and their carers; views and attitudes toward CM use; and perceived efficacy of treatment.

### Survey Design

The survey instrument was designed to take 20–30 min to complete and incorporated pre-existing validated tools, namely, Bakas Caregiving Outcomes Scale^©^ (16), PedsQL™ Eosinophilic Esophagitis Module Standard Version 3.0 Parent Reports^©^ (17), and PedsQL™ Infant Scales^©^ (18), along with other adapted survey items (see Table 1). In addition to the pre-existing instruments, the survey items were drafted to confirm eligibility [mandatory questions included: “Did your child have an endoscopy to assist with EoE diagnosis?” and “Has your child been diagnosed with EoE by a pediatric gastroenterologist (or other medical specialist)?”], ensure that the questions addressed the CM use in children, not the carer, and gauge treatment burden [the workload attributed to healthcare, and its impact on patient well-being and functioning (38)], and access to funding support for EoE patients. This included questions about access to private health insurance and a government-issued healthcare card and/or carer allowance. Australia's public health system provides access to a wide range of hospital and health services for all Australians at low or no cost (39). In Australia, additional private health insurance can be purchased to cover specific costs related to private hospital treatment and other medical services (40). Carer allowance is means tested and is available for those persons who provide additional daily care to a child who has a serious chronic illness (41). Healthcare cards can reduce the cost of certain prescription medications and medical doctor consultations and are issued to persons receiving various government payments or subsidies, including carer allowance (42).

**Table 1:** Validated tools incorporated in the survey instrument.

**Validated tool**	**Measures**
Bakas Caregiving Outcomes Scale^©^ Bakas (2007)^*^ (16)	Life changes in family caregivers. Validated for use in caregivers
PedsQL™ Eosinophilic Esophagitis Module Standard Version 3.0 Parent reports^©^ Varni (2012)^*^ (17)	Parents' perceptions of the HRQoL of their EoE child in the previous month. Validated for use in pediatric EoE for children aged 2–18 years old
PedsQL™ Infant Scales^©^ Varni (1998)^*^ (18)	HRQoL. Validated for use in healthy and ill infants aged 0–24 months
Australian Bureau of Statistics 2016 Census Household Form^#^ (19)	Age, ethnicity, and language spoken at home. Validated for use in the Australian general population
PedsQL™ Family Information Form^©^ Varni (1998)^#^ (20)	Demographic details including the child's date of birth and gender and the impact of EoE on hospital visits, school absences, and parental work absences. Validated for use in pediatric patients with chronic disease
Complementary Medicine Use, Literacy and Disclosure in the Australian Population^#^ (21, 22)	Patterns of CM use; understanding and communication of CM use. Validated for use in the Australian general population
Complementary Therapies Questionnaire^#^ (12)	Experiences and perceptions of CM use including concerns, reasons for use, views on future use, decisions leading to use, and perceived efficacy of CM treatment. Validated for use in pediatric inflammatory bowel disease patients

* Minor amendments were made, including spelling and grammatical changes, to ensure that they were appropriate for an Australian audience and were specific to EoE populations.

# Specific survey items have also been adopted from other pre-existing validated tools to confirm sociodemographic details (19, 20), patterns of CM use, understanding and communication of CM use patterns (21, 22), and experiences and perceptions of CM use including concerns, reasons for use, views on future use, decisions leading to use, and perceived efficacy of CM treatment (12).

*Additional survey items were developed from literature reporting CM and other health service use in chronic inflammatory pediatric diseases, including gastrointestinal disorders (12, 13, 17, 23–37), to confirm eligibility, ensure that that the questions addressed CM use in children not the career, gauge treatment burden [the workload attributed to healthcare and its impact on patient well-being and functioning (38)], and access funding support for EoE patients*.

The survey was tested for content and face validity, with feedback obtained by two parents of children with chronic disease using a paper version of the survey, followed by online testing *via* the Survey Gizmo® platform by the parent of a child with eosinophilic gastroenteritis. The lead researcher and the parent of a child with EoE also tested the online version on different devices (e.g., tablet, phone, and laptop). Minor changes to improve readability and understanding were made based on the feedback from the different parties and following discussions among the research team.

### Participants

The study participants were English-speaking carers of children with a confirmed EoE diagnosis (≤ 18 years of age) in Australia. The target survey sample size of 210 parents of EoE children was determined to achieve a 95% confidence level, confidence interval of 5, and population of 462 from a prevalence rate of 1 in 10,000 (2, 43).

### Recruitment

Purposive convenience and snowball sampling were employed. The responses were limited to one survey per family; if more than one child in the family had EoE, the respondents were asked to complete the survey for the eldest child only.

The Australian pediatric EoE support network, AusEE Inc., promoted the survey to their network of consumer members, their medical advisory board, other specialist doctors, and organizations such as Allergy and Anaphylaxis Australia and Allergy and Immunology Foundation of Australasia. Professional associations—the Australasian Society of Clinical Immunology and Allergy, the Gastroenterological Society of Australia, Australian Society of Pediatric Gastroenterology Hepatology and Nutrition, and the Royal Australian College of General Practitioners—invited their members to assist with the recruitment. The research team also directly contacted specialized EoE clinicians, general practitioners (GPs), and hospital-based pediatric allergy and gastroenterology departments across Australia and invited their assistance with recruitment. Snowball sampling was used to encourage medical specialists and carers of pediatric EoE children to ask others to participate. The survey incorporated a participant information sheet and a consent statement, with consent implied by survey completion. The participants had the opportunity to win one of 10 AU$50 gift vouchers (*via two* prize draws of five vouchers each) upon survey completion.

### Data Collection

#### Demographic Characteristics

Child age, age at diagnosis, gender, ethnicity, residential postcode, health cover, and carer allowance details were obtained, as well as carer gender and their relationship to the EoE child.

#### Health Service and Treatment Utilization

The participants were asked to provide information regarding the health services and treatment used by their EoE child, including the recommendation source of each health service and treatment and the frequency of practitioner consultations and associated out-of-pocket expenditure in the previous 12 months. Medicine use, treatments, and practices were separated into pharmaceuticals, CM products, CM therapies, and dietary changes. In accordance with schedule 14 of the Australian Government Therapeutic Goods Regulations 1990, CM products were defined according to their active ingredient, e.g., “a vitamin or provitamin,” not by the purpose of usage, i.e., a vitamin deficiency (44).

### Data Analysis

Descriptive statistics were determined for each variable. STATA/IC 15 statistical analysis software was used for the data analysis. Missing answers for questions where the respondents were asked to indicate agreement and no other option was provided were classified as “no.” All other instances where an answer was not provided were excluded from the analysis. Potential overlap of practitioner type was identified through the participants who provided examples of one practitioner with multiple qualifications, including allergist/immunologist (*n* = 3), dietitian/nutritionist (*n* = 1), and naturopath/nutritionist (*n* = 1). As there was no way to determine if multiple answers were selected for the same practitioner for all respondents, original values were retained. “Other” open-text responses in all categories were reviewed and amendments were made accordingly. For example, where not already allocated to another practitioner, “undergoing hypnotherapy” was allocated to a hypnotherapist, and “once” was reclassified as one to two visits to a practitioner. Where the respondents provided “Other” recommendation sources for child medicines, products, and therapies, that were among the listed options of specified practitioners, the “Other” response was re-classified to the listed category. Where gastroenterologist was listed as “Other,” the response was amended to pediatric gastroenterologist. The frequency of visit percentages was calculated from individual “use ever” totals for each health practitioner type. Only respondents who indicated that the use of the medicine, product, and therapy type was for their child's EoE were included in the data analysis. A new variable was also created to represent the total number of practitioner types visited, excluding visits with a “pharmacy or health food store assistant” as this role does not require defined professional or clinical training. Poisson regression analysis was used to determine the relationship between ‘time since diagnosis' and use of treatments that have been strongly recommended against (in this study, mast cell stabilizers and antihistamines) (5). Logistic regression analysis was used to determine if private health insurance status was a predictor of CM use for pediatric EoE.

## Results

A total of 181 survey responses were included in the analysis after the incomplete survey responses were removed.

### Demographics

A total of 232 survey responses were received. Thirty-five incomplete responses were removed, as well as those that did not meet the inclusion criteria (*n* = 16). These included responses with no confirmed EoE diagnosis (*n* = 12), residence outside of Australia (*n* = 1), completion by an EoE child instead of their parent (*n* = 2), and child whose age is over 18 years (*n* = 1). The remaining 181 responses were used for data analysis.

### Carer and EoE Child Characteristics

The surveys were almost exclusively completed by a parent (*n* = 178)—in most cases, the mother (*n* = 173) of the EoE child (see Table 2). The children were identified predominantly as White/Caucasian (93.3%) and males (71.7%), ages between 13 months and 18 years were represented (mean 9.70; SD 4.67), and the mean time since diagnosis for this study was 4.13 years (SD 3.38; min 0; max 14.17). Most children were covered by a private health insurance (63.3%), but <1/2 of the families received additional financial support from the Australian Government to reduce out-of-pocket medical expenses through a healthcare card (39.4%) or financial support through a carer allowance (27.8%). Private health insurance status was not found to be a significant predictor of overall CM use (practitioner, product, or therapy) for pediatric EoE (*p* = 0.86, OR 1.06; 95% CI: 0.54–2.09), CM practitioner use only (*p* = 0.19, OR 1.54; 95% CI: 0.81–2.94), or CM product or therapy use only (*p* = 0.92, OR 0.97; 95% CI: 0.51–1.85).

**Table 2:** Sociodemographic characteristics of carers and of EoE children as reported by carers.

**Child's characteristics**	***n* (%)**	**Carer characteristics**	***n* (%)**
Child's gender (*n* = 180)		Carer gender (*n* = 180)	
Male	129 (71.7)	Male	5 (2.8)
Female	51 (28.3)	Female	175 (97.2)
Child's age^#^ (*n* = 180)		Carer relationship to child (*n* = 179)	
1–23 months	4 (2.2)	Mother	173 (96.6)
2–4 years	22 (12.2)	Father	5 (2.8)
5–7 years	35 (19.4)	Grandmother	1 (0.6)
8–12 years	69 (38.3)	Carer state of residence (*n* = 179)	
13–18 years	50 (27.8)	Australian Capital Territory	4 (2.2)
Child's age at diagnosis (*n* = 181)		New South Wales	61 (34.1)
1–23 months	45 (24.9)	Northern Territory	1 (0.6)
2–4 years	46 (25.4)	Queensland	46 (25.7)
5–7 years	34 (18.8)	South Australia	14 (7.8)
8–12 years	45 (24.9)	Tasmania	0 (0.0)
13–18 years	11 (6.1)	Victoria	31 (17.3)
Time since diagnosis (*n* = 180)	SD; min–max	Western Australia	22(12.3)
Mean 4.13 years	3.38; 0–14.17	Applied for and approved carer allowance (*n* = 180)	
Child's ethnicity (*n* = 180)	*n* (%)	Yes	50 (27.8)
White/Caucasian	168 (93.3)	No	130 (72.2)
Aboriginal/Torres strait islander	1 (0.6)		
Asian	4 (2.2)		
Middle eastern	2 (1.1)		
Other	5 (2.8)		
Current healthcare card (*n* = 180)			
Yes	71 (39.4)		
No	109 (60.6)		
Current private health insurance (*n* = 180)			
Yes	114 (63.3)		
No	61 (33.9)		
Unsure	5 (2.8)		

# *Child's age at survey completion*.

### Health Service Use

Most children (88.4%) received care from three or more different types of practitioner, with almost two-thirds (60.8%) consulting six or more practitioner types for their EoE. Most children (91.2%) had consulted a medical doctor for their EoE. The most commonly accessed healthcare practitioners at any time for EoE were a pediatric gastroenterologist (86.2%), GP (84.5%), allergist (70.2%), and dietitian (69.6%) (reported in Table 3). Almost half of the respondents had consulted a CM practitioner (40.3%) at some time-point after their child's diagnosis, with a naturopath (22.1%) being the most commonly accessed. Most respondents indicated that they saw any type of medical doctor once or twice in the last 12 months. GPs were mostly visited, with more than six visits in the past 12 months (27.0%). Although we are unable to determine if multiple practitioner use was simultaneous or sequential in the previous 12 months, the mean number of different types of practitioner seen for a child's EoE was 4.6 (SD 2.99; min 0; max 12); one-fifth (20.7%) of the respondents indicated that their child had seen both a medical doctor and a CM practitioner.

**Table 3:** Prevalence and frequency of health service use by EoE children (*n* = 181).

**Practitioner type**	**Use ever** ***n* (%)^**#**^**	**Frequency of visits in the past 12 months** ***n*** **(%)**				
		**None**	**1–2**	**3–4**	**5–6**	**More than 6**
		***n* (%)**	***n* (%)**	***n* (%)**	***n* (%)**	***n* (%)**
**MEDICAL DOCTORS**						
Allergist	127 (70.2)	31 (25.4)	69 (56.5)	14 (11.5)	5 (4.1)	3 (2.5)
General practitioner	153 (84.5)	20 (13.5)	44 (29.7)	31 (21.0)	13 (8.8)	40 (27.0)
Hospital doctor	99 (54.7)	28 (29.8)	30 (31.9)	15 (16.0)	13 (13.8)	8 (8.5)
Immunologist	90 (49.7)	19 (22.9)	47 (56.6)	10 (12.1)	5 (6.0)	2 (2.4)
Pediatric gastroenterologist	156 (86.2)	9 (6.1)	55 (37.2)	45 (30.4)	32 (21.6)	7 (4.7)
Pediatrician	96 (53.0)	34 (37.4)	42 (46.1)	9 (9.9)	4 (4.4)	2 (2.2)
**CM PRACTITIONERS**						
Acupuncturist	7 (3.9)	5 (71.4)	1 (14.3)	1 (14.3)	0 (0.0)	0 (0.0)
Aromatherapist	3 (1.7)	1 (33.4)	1 (33.3)	1 (33.3)	0 (0.0)	0 (0.0)
Chiropractor	31 (17.1)	13 (46.4)	4 (14.3)	7 (25.0)	0 (0.0)	4 (14.3)
Homeopath	11 (6.1)	5 (50.0)	1 (10.0)	3 (30.0)	1 (10.0)	0 (0.0)
Massage therapist	8 (4.4)	2 (28.6)	2 (28.6)	1 (14.2)	2 (28.6)	0 (0.0)
Naturopath	40 (22.1)	17 (46.0)	11 (29.7)	4 (10.8)	0 (0.0)	5 (13.5)
Osteopath	8 (4.4)	6 (85.7)	0 (0.0)	1 (14.3)	0 (0.0)	0 (0.0)
Relaxation/meditation teacher	7 (3.9)	2 (33.3)	4 (66.7)	0 (0.0)	0 (0.0)	0 (0.0)
Tai chi or qigong teacher	1 (0.6)	1 (100.0)	0 (0.0)	0 (0.0)	0 (0.0)	0 (0.0)
Traditional Chinese medicine practitioner	1 (0.6)	1 (100.0)	0 (0.0)	0 (0.0)	0 (0.0)	0 (0.0)
Western herbalist	4 (2.2)	3 (75.0)	0 (0.0)	0 (0.0)	0 (0.0)	1 (25.0)
Yoga teacher	4 (2.2)	0 (0.0)	2 (66.7)	1 (33.3)	0 (0.0)	0 (0.0)
**OTHER HEALTH PRACTITIONERS OR HEALTH WORKERS**						
Counselor or other mental health worker	45 (24.9)	10 (23.8)	11 (26.2)	3 (7.1)	7 (16.7)	11 (26.2)
Dietitian	126 (69.6)	44 (36.7)	39 (32.5)	22 (18.3)	9 (7.5)	6 (5.0)
Nutritionist	41 (22.7)	17 (47.2)	12 (33.3)	2 (5.6)	1 (2.8)	4 (11.1)
Pharmacist	74 (40.9)	7 (10.0)	15 (21.4)	11 (15.7)	10 (14.3)	27 (38.6)
Pharmacy or health food store assistant	41 (22.7)	4 (10.5)	11 (28.9)	5 (13.2)	6 (15.8)	12 (31.6)
Other practitioner	20 (11.1)	2 (13.3)	3 (20.0)	2 (13.3)	1 (6.7)	7 (46.7)

# *The total number of practitioners is greater than the number of responses as some respondents listed more than one other practitioner*.

### Use and Perceived Effectiveness of Pharmaceuticals, CMs, and Dietary Changes

Pharmaceuticals (86.2%) were the most commonly used treatment option at any time for pediatric EoE, followed by dietary changes (78.5%), CM products (60.2%), and CM therapies (23.2%) (see Table 4). Most respondents indicated that reflux medications (77.9%) had been used for EoE management. Probiotics (43.1%) and nutritional supplements (40.9%) were the most used CM products. Dietary changes were common, with over three quarters (75.1%) of all respondents indicating that they had used elimination diets in the management of their child's EoE, followed by elemental formula (43.7%).

**Table 4:** Perceived effectiveness of pharmaceuticals, complementary medicines (CMs), and dietary changes for pediatric EoE (*n* = 181).

**Medicines, treatments, and practices**	**Use ever** ***n* (%)**	**Perceived effectiveness** ***n*** **(%)**				
		**Very effective**	**Partially effective**	**Not effective**	**Made worse**	**Unsure**
		***n* (%)**	***n* (%)**	***n* (%)**	***n* (%)**	***n* (%)**
**PHARMACEUTICALS**						
Antihistamines	107 (59.1)	15 (15.5)	39 (40.2)	24 (24.7)	2 (2.1)	17 (17.5)
Corticosteroids	127 (70.2)	42 (35.9)	45 (38.5)	15 (12.8)	3 (2.6)	12 (10.2)
Mast cell stabilizers	23 (12.7)	8 (42.1)	5 (26.4)	2 (10.5)	2 (10.5)	2 (10.5)
Reflux medications	141 (77.9)	41 (30.8)	45 (33.8)	31 (23.3)	3 (2.3)	13 (9.8)
Other pharmaceuticals^∧^	2 (1.2)					
Any pharmaceuticals^∧^	156 (86.2)					
**CM PRODUCTS**						
Chinese herbal medicines	4 (2.2)	0 (0.0)	3 (75.0)	0 (0.0)	1 (25.0)	0 (0.0)
Flower essences	14 (7.7)	1 (8.3)	1 (8.3)	5 (41.7)	0 (0.0)	5 (41.7)
Homeopathic medicines	16 (8.8)	0 (0.0)	6 (46.1)	4 (30.8)	2 (15.4)	1 (7.7)
Nutritional supplements	74 (40.9)	6 (9.1)	24 (36.4)	14 (21.2)	2 (3.0)	20 (30.3)
Probiotics	78 (43.1)	5 (7.3)	21 (30.4)	17 (24.6)	6 (8.7)	20 (29.0)
Western herbal medicines	7 (3.9)	2 (28.6)	3 (42.8)	2 (28.6)	0 (0.0)	0 (0.0)
Other complementary medicines^∧^	3 (1.7)					
Any complementary medicines^∧^	109 (60.2)					
**CM THERAPIES**						
Acupuncture	6 (3.3)	0 (0.0)	1 (16.7)	2 (33.3)	0 (0.0)	3 (50.0)
Aromatherapy	18 (9.9)	1 (6.7)	6 (40.0)	3 (20.0)	0 (0.0)	5 (33.3)
Massage	12 (6.6)	0 (0.0)	4 (40.0)	4 (40.0)	0 (0.0)	2 (20.0)
Relaxation techniques/meditation	25 (13.8)	1 (4.8)	13 (61.9)	7 (33.3)	0 (0.0)	0 (0.0)
Yoga	5 (2.8)	0 (0.0)	3 (75.0)	1 (25.0)	0 (0.0)	0 (0.0)
Other complementary treatments^∧^	6 (3.3)					
Any complementary treatment^∧^	42 (23.2)					
**DIETARY CHANGES**						
Elemental formula	79 (43.7)	34 (46.6)	18 (24.6)	14 (19.2)	0 (0.0)	7 (9.6)
Elimination diet	136 (75.1)	46 (36.5)	50 (39.7)	14 (11.1)	11 (8.7)	5 (4.0)
Other dietary changes^∧^	2 (1.1)					
Any dietary changes^∧^	142 (78.5)					

∧ *Perceived effectiveness not available as in some cases one option was chosen for multiple “other” medicines, treatments, and practices*.

Amongst the pharmaceuticals listed, most respondents (74.4%) perceived corticosteroids as effective, followed by mast cell stabilizers (68.5%). The mast cell stabilizers (*n* = 23) also had the highest percentage (10.5%) of “made worse” responses, over four times greater than each of the other pharmaceuticals. Poisson regression analysis determined that the risk of children using treatments strongly recommended against in EoE therapy, namely, mast cell stabilizers and antihistamines, is 1.6 times greater (CI 1.0–2.6, *p* = 0.05) between 2 and 4 years since diagnosis and 1.9 times greater (CI 1.2–2.9, *p* = 0.003) at 4 years or more after diagnosis when compared with children in their first 2 years since diagnosis. Almost one quarter of the respondents perceived reflux medications (*n* = 31) to be ineffective in EoE management. Despite the small sample sizes for most CM products, the respondents reported high levels of perceived effectiveness for Chinese herbal medicines (*n* = 3, 75.0%) and Western herbal medicines (*n* = 5, 71.4%). Acupuncture was more often perceived to be ineffective (*n* = 2; 33.3%) than effective (*n* = 1; 16.7%). No CM therapy was perceived to have made the child's EoE worse. While most respondents felt that elemental formula was effective (71.2%), 14 respondents found it ineffective and seven were uncertain. The overall effectiveness of the elimination diet (76.2%) was slightly higher than that of the elemental formula.

### Sources of Recommendation

All pharmaceuticals used for EoE treatment were predominantly recommended by medical doctors (Table 5). Corticosteroids were recommended by a pediatric gastroenterologist in over 80% of cases, as were reflux medications (87.9%). Mast cell stabilizers were only recommended by immunologists, pediatric gastroenterologists, and allergists. All CM therapies were predominantly self-prescribed by the carer (Table 5). Medical doctors and other non-CM practitioners were more likely to recommend nutritional supplements and probiotics than any other type of CM. Dietitians recommended nutritional supplements in over 40% of cases, while only 28.4% (*n* = 21) were recommended by pediatric gastroenterologists and 24.3% (*n* = 18) were recommended by CM practitioners. Probiotics were equally recommended by CM practitioners (25.6%) or self-prescribed (25.6%). Elimination diet (69.9%) and elemental formula (62.0%) were mostly recommended by pediatric gastroenterologists, followed by allergists (elimination diet: 34.6%, elemental formula: 31.7%) and dieticians (elimination diet: 30.2%, elemental formula: 29.1%). Some patients who had used an elimination diet had never seen a dietician or nutritionist (15.4%). Almost one-third of carers self-prescribed (29.4%, *n* = 47/160) non-prescription only pharmaceuticals, CMs, or dietary changes for their EoE child.

**Table 5:** Source of recommendation for pharmaceuticals, complementary medicines (CMs), and dietary changes for pediatric EoE (*n* = 181).

**Pharmaceuticals, CMs, and dietary changes**	**Source of recommendation** ***n*** (%)^*^												
	**Allergist**	**Immunologist**	**Pediatric gastro-enterologist**	**Pediatrician**	**General practitioner**	**Hospital doctor**	**Pharmacist**	**Dietitian or nutritionist**	**Counselor or other mental healthcare worker**	**CM^**∧**^ practitioner**	**Pharmacy or health food store assistant**	**Family or friend**	**Self-prescribed**
	***n* (%)**	***n* (%)**	***n* (%)**	***n* (%)**	***n* (%)**	***n* (%)**	***n* (%)**	***n* (%)**	***n* (%)**	***n* (%)**	***n* (%)**	***n* (%)**	***n* (%)**
**PHARMACEUTICALS**													
Antihistamines (*n* = 107)	54 (50.5)	29 (27.1)	24 (22.4)	12 (11.2)	32 (29.9)	8 (7.5)	4 (3.7)	2 (1.9)	0 (0.0)	1 (0.9)	0 (0.0)	3 (2.8)	4 (3.7)
Corticosteroids (*n* = 127)	22 (17.3)	13 (10.2)	102 (80.3)	7 (5.5)	7 (5.5)	6 (4.7)	0 (0.0)	0 (0.0)	0 (0.0)	0 (0.0)	0 (0.0)	0 (0.0)	0 (0.0)
Mast cell stabilizers (*n* = 23)	6 (26.1)	10 (43.5)	9 (39.1)	0 (0.0)	0 (0.0)	0 (0.0)	0 (0.0)	0 (0.0)	0 (0.0)	0 (0.0)	0 (0.0)	0 (0.0)	0 (0.0)
Reflux medications (*n* = 141)	21 (14.9)	11 (7.8)	124 (87.9)	21 (14.9)	22 (15.6)	3 (2.1)	0 (0.0)	1 (8.3)	0 (0.0)	0 (0.0)	0 (0.0)	0 (0.0)	0 (0.0)
**CM PRODUCTS**													
Chinese herbal medicines (*n* = 4)	0 (0.0)	0 (0.0)	0 (0.0)	0 (0.0)	0 (0.0)	0 (0.0)	0 (0.0)	0 (0.0)	0 (0.0)	3 (75.0)	0 (0.0)	1 (25.0)	0 (0.0)
Flower essences (*n* = 14)	1 (7.1)	0 (0.0)	0 (0.0)	0 (0.0)	1 (7.1)	0 (0.0)	1 (7.1)	1 (7.1)	0 (0.0)	5 (35.7)	0 (0.0)	1 (7.1)	8 (57.1)
Homeopathic medicines (*n* = 16)	0 (0.0)	1 (6.3)	0 (0.0)	0 (0.0)	0 (0.0)	0 (0.0)	1 (6.3)	0 (0.0)	0 (0.0)	9(56.3)	0 (0.0)	2 (12.5)	2 (12.5)
Nutritional supplements (*n* = 74)	10 (13.5)	6 (8.1)	21 (28.4)	4 (5.4)	10 (13.5)	1 (1.4)	2 (2.7)	33 (4.6)	0 (0.0)	18 (24.3)	1 (1.4)	1 (1.4)	10 (13.5)
Probiotics (*n* = 78)	7 (9.0)	6 (7.7)	11 (14.1)	3 (3.9)	15 (19.2)	0 (0.0)	4 (5.1)	17 (21.8)	0 (0.0)	20 (25.6)	2 (2.6)	2 (2.6)	20 (25.6)
Western herbal medicines (*n* = 7)	0 (0.0)	0 (0.0)	0 (0.0)	0 (0.0)	0 (0.0)	0 (0.0)	1 (14.3)	0 (0.0)	0 (0.0)	5 (71.4)	0 (0.0)	2 (28.6)	1 (14.3)
**CM THERAPIES**													
Acupuncture (*n* = 6)	0 (0.0)	1 (16.7)	0 (0.0)	0 (0.0)	0 (0.0)	0 (0.0)	0 (0.0)	0 (0.0)	0 (0.0)	2 (33.3)	0 (0.0)	0 (0.0)	4 (66.7)
Aromatherapy (*n* = 18)	0 (0.0)	0 (0.0)	0 (0.0)	1 (5.6)	0 (0.0)	0 (0.0)	0 (0.0)	0 (0.0)	0 (0.0)	7 (38.9)	1 (5.6)	3 (16.7)	6 (33.3)
Massage (*n* = 12)	0 (0.0)	0 (0.0)	0 (0.0)	1 (8.3)	1 (8.3)	0 (0.0)	1 (8.3)	0 (0.0)	1 (8.3)	3 (25.0)	0 (0.0)	0 (0.0)	3 (25.0)
Relaxation techniques/Meditation (*n* = 25)	1 (4.0)	0 (0.0)	1 (4.0)	1 (4.0)	2 (8.0)	1 (4.0)	0 (0.0)	0 (0.0)	7 (28.0)	2 (8.0)	0 (0.0)	0 (0.0)	11 (44.0)
Yoga (*n* = 5)	0 (0.0)	0 (0.0)	0 (0.0)	0 (0.0)	1 (20.0)	1 (20.0)	0 (0.0)	0 (0.0)	1 (20.0)	0 (0.0)	0 (0.0)	0 (0.0)	2 (40.0)
**DIETARY CHANGES**													
Elemental formula (*n* = 79)	25 (31.7)	9 (11.4)	49 (62.0)	9 (11.4)	4 (5.1)	3 (3.8)	0 (0.0)	23 (29.1)	0 (0.0)	0 (0.0)	0 (0.0)	0 (0.0)	1 (1.3)
Elimination diet (*n* = 136)	47 (34.6)	21 (15.4)	95 (69.9)	10 (7.4)	3 (2.2)	3 (2.2)	0 (0.0)	42 (30.9)	0 (0.0)	7 (5.2)	0 (0.0)	0 (0.0)	6 (4.4)

* Total sources of recommendation may be different than the number of users if the respondents selected more than one or no recommendation source.

∧ *Includes acupuncturist, aromatherapist, chiropractor, homeopath, massage therapist, naturopath, osteopath, relaxation/meditation teacher, tai chi or qigong teacher, traditional Chinese medicine practitioner, Western herbalist, and yoga teacher*.

## Discussion

To our knowledge, this is the first study to explore health service, medicine, and CM use for pediatric EoE. It is difficult to estimate the percentage of the pediatric EoE population in Australia that was captured by this survey as the prevalence rates are changing rapidly (43). The survey was designed in 2017 and undertaken between September 2018 and March 2019. During this time, the prevalence data, based on international (4, 45) and Australian (43) studies, ranged from 1 to 5 in 10,000 and may be as high as 1 in 1,000 in 2020 (2). According to Australian census data (46), it would mean that the survey captured between 4 and 40% of the pediatric EoE population in Australia, depending on what would be considered as accurate prevalence data at the time. A 2018 systematic review and meta-analysis (47), which included 13 studies, focused on HRQoL in patients with EoE of all ages. The sample sizes ranged from *n* = 8 (Australia) (48) to *n* = 140 (USA) (49), emphasizing the large sample size of the study reported here. The EoE children in our study were identified predominantly as White/Caucasian (93.3%) and of male gender (71.7%), which is representative of the general pediatric EoE population (50). The representation by children of all ages (between 13 months to 18 years) and the broad range of time since diagnosis indicate that the responses represented patients at varied stages in their EoE management.

Our study showed that most (86.2%) children had been given a pharmaceutical at some stage to treat their EoE. Reflux medications such as proton pump inhibitors are a first-line treatment option for EoE (7) and were the most commonly used pharmaceutical. However, proton pump inhibitors may be associated with adverse side effects when used for a long term (51), and almost one quarter of those respondents who had used them perceived them to be ineffective in EoE management, which is in line with previous findings (52). Proton pump inhibitors can reduce the absorption and bioavailability of nutrients, such as calcium, iron, magnesium, and vitamin B12 (51), which are particularly important in a pediatric population (53). Yet there is limited information on the safety, benefits, and bioavailability of different forms of nutrients, specifically for supplementation in EoE. Mast cell stabilizers and antihistamines, although their use is strongly recommended against for EoE treatment (5), were perceived as effective by most responders. Yet 10.5% of the respondents perceived mast cell stabilizers to have made EoE symptoms worse, over four times greater than reported for each of the other pharmaceuticals. This reflects the need for qualitative interviews to further understand how efficacious treatment is perceived by the parents of children with EoE and for additional research to provide evidence-based treatment options for these patients as well as improved practitioner awareness and education regarding EoE treatment guidelines.

The reported CM use was high, with the respondents indicating that they had consulted with a CM practitioner (40.3%), used CM products (60.2%), or used CM therapies (23.2%) to manage their child's EoE. Nutritional supplements and probiotics were the CM products most commonly recommended by a health professional for EoE, with medical doctors and other non-CM practitioners being more likely to recommend them than any other CM product. As CM products were defined according to their active ingredient (e.g., “a vitamin or provitamin”), not by the purpose of usage (e.g., to correct a deficiency or to supplement in general), supplementation with, e.g., vitamin D, calcium, or iron to correct deficiencies is also counted as CM use. Anecdotally, the wait time for consultations within the Australian public health system [all costs are subsidized by the Australian Government for Australian citizens (39)] for pediatric gastroenterologists and pediatric allergy specialists (allergist/immunologist) can be 12–18 months. Although the waitlists may be reduced for patients opting to consult pediatric gastroenterologists in private practice, out-of-pocket expenses can be higher, particularly for those without a private health insurance cover. Long wait times to access pediatric allergy and gastroenterology specialists within the Australian public health system but easy access to a CM practitioner and products may enhance CM use in this population. Given that carers of children with chronic inflammatory gastrointestinal disease expect the practitioners to be knowledgeable about CM use (54), further research into commonly used CMs for pediatric EoE and education are required so that all practitioners involved in the care are enabled to give evidence-based advice.

The participants in our study reported perceiving some pharmaceuticals and CM products to lack efficacy or worsen symptoms. While there may be several reasons for these results, including worsening of symptoms due to the use of an ineffective treatment, they warrant further investigation in consumer interviews. Inadvertent exposure to an antigenic EoE or IgE allergy trigger can occur due to the inadequate health literacy of the carer, poorly executed elimination diet, or undisclosed excipient ingredients in the medicine itself. For example, otherwise effective medicines may be perceived as ineffective due to containing unknown excipients such as milk proteins, soy, wheat, corn, rice, and potato, which can be common EoE antigens (55). Depending on therapeutic regulations, this information may be omitted from product labeling (56, 57). It is therefore vital to raise awareness and knowledge among clinicians and self-prescribing carers of EoE children about medicine excipients and engage the expertise of pharmacists or other stakeholders to reduce the risk of exposure to known EoE triggers.

Our study also found that almost one-third of carers self-prescribe non-prescription-only pharmaceuticals, CMs or dietary changes for their EoE child. The importance of carers in the management of pediatric EoE and the selection of treatment options should not be underestimated. Consequently, practitioners should facilitate open discussions with carers regarding their complete medicine and treatment use for their EoE child. With limited research into the efficacy and safety of EoE treatment and management options, parent perceptions, experiences, and decisions provide valuable insights (58), which are worthy of increased attention. Parental proxy report in young children with EoE can function as an adequate marker for child self-reported symptoms and HRQoL measures (59). Additionally, parent involvement in decision making has been shown to improve a child's treatment outcomes (60), suggesting that carers play an important role in disease management and should be seen as treatment partners by the practitioners. However, due to the scarcity of evidence-based treatment options in EoE, it is challenging for the practitioners to effectively fulfill the carers' and the patients' expectations and needs.

Elimination diets were commonly used by study participants and were mostly reported as being recommended by pediatric gastroenterologists. Decisions surrounding the choice and the implementation of dietary elimination and re-introduction are complex and can result in treatment failure or symptom worsening, potentially due to factors such as inadequate patient education, non-adherence, and atypical individual triggers (61, 62). Unfortunately, at least one in seven respondents who had used an elimination diet had never consulted a dietitian or nutritionist. The reason may lay in the fact that neither profession is classified as a registered health profession in Australia (63), leading to a lack of clarity surrounding the education, qualification, and professional standards of these professions for consumers. As EoE is one of many diseases requiring expert dietary management, it is imperative that these professions are regulated through professional registration which would result in the implementation of mandatory educational and practice standards, leading to enhanced trust and acceptance by consumers and therefore most likely to higher consultation rates. There is evidence that gastroenterologists often agree to patient-driven elimination diets without dietitian support and do not adhere to the recommendations for repeated biopsies to monitor ongoing response to therapy (64). This underutilization of dietitians and nutritionists in our study may reflect a lack of referral by gastroenterologists, allergists, and immunologists or a scarcity of practitioners with specialized knowledge; hence, identifying and accessing them may prove challenging for both the referring practitioners and the carers. Given that elimination diets are first-line treatment options in EoE and the high percentage of EoE patients using them, it is paramount to increase workforce education and educational resources and encourage collaboration between all practitioners to establish a wider referral network and provide specified support for EoE patients.

The necessity of collaboration between practitioners and the close communication with carers is particularly warranted as most children received care from three or more different practitioner types, with almost two-thirds having seen six or more different types of practitioner for their EoE. This high rate of diversity in practitioner types accessed for children with EoE is congruent with existing data on healthcare utilization by children with a rare disease (65). The parents of children with a rare disease often feel isolated and under-supported and perceive that there is poor coordination between care providers, requiring the parent to fill multiple roles and become the “expert” in the care of their child (65, 66). This social burden may be amplified by the financial burden to carers. In Australia, private health insurance can cover specific cost related to private hospital treatment and other medical services, which could include certain CMs (40). Our study shows that a higher percentage of EoE patients (63.3%) have private health insurance than is seen in the general Australian population (53.5%) (67). Carer allowance is available for those persons who provide additional daily care to a child who has a serious chronic illness (41). Healthcare cards can reduce the cost of certain prescription medications and medical doctor consultations and are automatically issued to those persons receiving various government payments or subsidies, including carer allowance (42). Our study showed that nearly two-thirds of the children did not have a healthcare card and a third of the respondents did not have private health insurance to reduce out-of-pocket expenses. Further exploration of financial burden is needed as existing data indicate that EoE-related costs are striking and consistently higher than those of healthy consumers (10, 47). The economic impact of poorly coordinated care encompassing the possible duplication of services as parents attempt to meet the healthcare needs of their family must be carefully considered. Collaboration between healthcare practitioners is thus even more important as it can help to identify the areas of unnecessary expenditure for patients and reduce financial barriers to treatment.

There are several study limitations. Only respondents involving a child diagnosed with EoE who had undergone an endoscopy were included in the analysis. An endoscopy is predominantly performed by a pediatric gastroenterologist; however, not all respondents indicated that their child had seen a pediatric gastroenterologist (or other medical doctor) for their EoE. This may be reflective of some respondents including only consultations post-EoE diagnosis and results in perceived lower numbers of pediatric gastroenterologist and other practitioner consultations as they occurred prior to or during the diagnosis process. Additionally, as the question regarding practitioner use ever required respondents to indicate agreement and no other option was provided, missing answers were classified as “no.” Therefore, practitioner visits may be under-reported due to missing answers. This study was based on self-reports and may therefore be subject to recall bias. Questions surrounding the efficacy of medications and therapies are perceptions of the carer only and may not be a true reflection of histological change in EoE.

## Conclusions

This study identified a large variety of practitioners who are involved in the care of EoE patients, resulting in a diverse range of treatment options being recommended and accessed and in a possible treatment-related burden. In addition, carer involvement in the choice of treatment for pediatric EoE is high. Referrals and collaboration between healthcare providers as well as education and shared decision making with carers are required to successfully navigate this complex disease and provide adequate care for children with EoE. The high rate of CM use, particularly given the absence of EoE guidelines in Australia, warrants further attention by clinicians, policy makers, and researchers.

## Data Availability Statement

The research data are stored securely as per Griffith University ethics approval and cannot be made publicly available. The authors will consider any reasonable request for access to the anonymized data according to the privacy statement provided with information and consent materials. Please direct any requests to the corresponding author.

## Ethics Statement

The studies involving human participants were reviewed and approved by Griffith University Human Research Ethics Committee (#2018/120). The patients/participants provided their written informed consent to participate in this study.

## Author Contributions

NH drafted the manuscript. All authors contributed to the study design, data analysis, and interpretation, provided editorial comments, read, and approved the final manuscript.

## Conflict of Interest

The authors declare that the research was conducted in the absence of any commercial or financial relationships that could be construed as a potential conflict of interest.
